# Fresh embryo transfer in the cleavage and blastocyst stages and pregnancy outcomes: A retrospective cross-sectional study

**DOI:** 10.18502/ijrm.v21i5.13477

**Published:** 2023-05-12

**Authors:** Seyedeh Mahsa Poormoosavi, Mohammad Amin Behmanesh, Sara Aryannejad, Sima Janati

**Affiliations:** ^1^Department of Histology, School of Medicine, Research and Clinical Center for Infertility, Dezful University of Medical Sciences, Dezful, Iran.; ^2^Department of Histology, School of Medicine, Dezful University of Medical Sciences, Dezful, Iran.; ^3^School of Medicine, Dezful University of Medical Sciences, Dezful, Iran.; ^4^Research and Clinical Center for Infertility, Ganjavian Hospital, Dezful-Andymeshk Road, Dezful, Iran.

**Keywords:** Embryo transfer, Cleavage, Blastocyst, Pregnancy outcomes.

## Abstract

**Background:**

Advances in cell culture media have led to a trend of embryo transfer from the early cleavage stages to blastocyst stage.

**Objective:**

The study aims to compare the effects of fresh embryo transfer in cleavage and blastocyst stage on pregnancy outcomes.

**Materials and Methods:**

This cross-sectional study was conducted on 1422 cases referred to the Umm-al-Banin Infertility Clinic Center, Dezful, Iran, between July 2013 and December 2020 who were candidates for in vitro fertilization/intracytoplasmic sperm injection for fresh embryo transfer. A total of 1246 cases were divided into 4 categories on days 2-5 or 6. Chemical and clinical pregnancy, abortion, multifetal pregnancy, ongoing pregnancy, and live birth rates were analyzed.

**Results:**

Fresh embryo transfer was performed in 28.5% of the cases on the 2 ${}^{\text{nd}}$ day, 45.8% on the 3 ${}^{\text{rd}}$ , 15.3% on the 4 ${}^{\text{th}}$ day, and 10.4% on days 5 or 6. The overall clinical pregnancy and live birth rates were estimated at 20.6% and 17.6% in the cleavage, and 17% and 14% in the blastocyst stages, respectively. However, no significant difference was observed in either group. In addition, there was no significant difference between groups in terms of the abortion, multifetal pregnancy, and ongoing pregnancy rate (p > 0.05).

**Conclusion:**

According to the results, the consequences of pregnancy in fresh embryo transfer at the blastocyst stage were not superior to embryo transfer at different stages of the cleavage process.

## 1. Introduction

Infertility associated with reproductive disorders is a pervasive problem in human societies, defined as the absence of pregnancy after 1 yr of regular intercourse without using contraception (1). In recent decades, the increasing demand of infertile couples for fertility has urged the development of various fertility technologies in treating fertility in vitro.

Embryo transfer and subsequent in vitro fertilization (IVF) are classically done at the cleavage on day 2 or 3 after fertilization, which is in the cell stage or the blastocyst stage, on days 5-6 (2). On the other hand, better pregnancy outcomes could be attained by defining another standard for the simultaneous transfer of more than one embryo, resulting in multiple births, along with consequences such as abortion or premature birth (3).

Evidence suggests that if the blastocyst is transferred to the uterus instead of the embryo in the cleavage stage, as developmental stages are fully monitored in the laboratory, the possibility of synchronizing the uterus to accept the embryo will further increase (3). On the other hand it is very similar to a natural phase and allows embryo self-selection after stimulation of the embryonic genome on day 3 (4). Current advances in cell culture media have conducted to a shift in the early embryo whereas transfer process from the cleavage stage to the blastocyst stage in IVF (3, 4). A recent study has indicated that since the morphological grade of the embryo in the cleavage and blastocyst stages cannot show chromosomal abnormalities; therefore, embryos with chromosomal defects may also progress to the blastocyst stage (5). Despite good reports on the prognosis of blastocyst embryo transfer (BET) programs, one study has shown that prolonged embryo culture, which can precede to a number of embryos failure to blastocyst stage, in vitro culture beyond embryonic genomic activation caused epigenetic effects on the embryo, and also an increased rate of transfer cessation and risk of monozygotic twins (4).

Embryo transfer was conventionally performed 2 days following oocyte retrieval, while changes in cell culture media allow embryos to be kept for longer times Delayed transfer from the 2 ${}^{\text{nd}}$ to the 3 ${}^{\text{rd}}$ day allows more fetal growth in vitro and may positively influence pregnancy outcomes (6). However, more retrospective and prospective studies are required to determine whether 2, 3, or 5-day-old embryo transfers differ in pregnancy outcomes.

Due to the contradictions in various studies regarding the consequences of embryo transfer in the cleavage and blastocyst stages, the present study aimed to retrospectively assess the records of the cases referred for embryo transfer to Dezful Infertility Center within the past 7 yr and review the outcomes of fresh embryo transfer in different stages of development.

## 2. Materials and Methods 

### Study design and participants 

In this cross-sectional study, the files of 1246 women who were candidates for IVF/intracytoplasmic sperm injection (ICSI) processes in the Umm Al-Banin Infertility Center in Ganjavian hospital, Dezful, Iran between July 2013 and December 2020 were studied.

The required data were collected by extracting information from the files of the participants based on the stage of the transferred embryo and completing checklists. The embryos of these individuals were transferred on days 2, 3, 4, and 5 or 6, depending on the policy of the infertility center. The pattern used in the study was based on the Strobe guideline checklist.

The participants aged between 20 and 43 yr were scheduled for fresh embryo transfers. Ladies with endocrine and metabolic condition; follicle-stimulating hormone concentration of > 12, uterine myoma, hydrosalpinges, endometriosis (grades III, IV); any surgery or defect in the uterine cavity, ovarian hyperstimulation syndrome, estradiol concentration ≥ 3500, obtained oocytes number > 15, no embryo development, ≥ 3 experience prior to failed IVF, empty follicle syndrome, and severe male factor were excluded from the study.

### Ovarian stimulation and oocyte retrieval

Using the antagonist protocol, controlled ovarian stimulation (COS) was performed as per the conventional method (7). Oocyte retrieval was done transvaginally 36 hr later subcutaneously injecting 250 µg of recombinant human chorionic gonadotropin (rHCG) vial (Ovitrelle, Merck-Serono, Germany) under ultrasound guidance.

Following oocytes collection, oocytes were denuded and incubated as a traditional method. Then denuded oocytes were verified for the nuclear status. The oocytes were scored via an inverted microscope (Olympus, Japan) (7). The oocytes were inseminated and subsequently incubated in the culture media. They were then assessed to confirm the show of pronuclei and fertilization 16-18 hr after using a Nikon inverted microscope (Olympus, Japan).

### Embryo grading, embryo transfer procedure

An expert embryologist evaluated the embryo quality, and the embryos for transfer were chosen in the opinion of morphologic features. Embryos were scored according to Hill's standards (8). On day 5, the blastocyst scoring system was used according to the standards of Gardner and School craft. This is centered on the creation and scale of expansion from early to totally expanded blastocyst of the blastocoele, the progress of the inner cell mass, and trophectoderm. The pooled score varied from 1-4 (A-D), with greater scores representing bad quality; embryos with a score of 4 (D) were not transferred (9).

After embryo grading, based on the embryo quality, age of women, and previous history of IVF failure, the number of transferred embryos was determined between 1 and 3. According to the clinics policy, embryo transfer at the blastocysts stages are performed at the request of the couple when there are at least 6 grade A/B embryos, severe male factor is absent, and patient has no history of more than 3 unsuccessful embryo transfers.

Fresh embryo transfer was performed 48-144 hr after ovum pick up by transabdominal ultrasonography guide by the single gynecologist with the same catheter as conventional method (10).

Participants were divided into 4 groups based on the stage of the transferred embryo. The first group of embryos was in the 2-day stage (group 1), the second group of 3-day embryos (group 2), the third group of 4-day embryos (group 3), and the 4 ${}^{\text{th}}$ group of 5-6 days' embryos (group 4). All groups were compared in terms of the results. In cases where progesterone was prescribed intramuscularly at 100 mg/day, it was initiated on the day of ovum pick up, and it was continued up to the 10 ${}^{\text{th}}$ wk of pregnancy as luteal support.

### Outcome measures 

The determined outcomes contained a chemical pregnancy rate, which was considered as a serum beta human chorionic gonadotropin level > 25 IU/ml 14 days next to the embryo transfer. The clinical pregnancy rate was verified by the number of gestational sacs with the fetal heartbeat on vaginal ultrasound 2 wk after a positive pregnancy test. The miscarriage rate was termed as the failure of pregnancy below 20 wk of gestation, and ongoing pregnancy was defined as pregnancy progressing further than ≥ 20 wk of gestation.

Additionally, participants data were also extracted from their files, including the cause of infertility, estradiol levels, number of total and mature oocytes, number and quality of the transferred embryos, chemical and clinical pregnancy rate, miscarriage rate, ongoing pregnancy rate, rate of live births, and multifetal pregnancies.

### Ethical considerations

Prior to starting the IVF/ICSI process, the couples signed the informed consent forms. The study was approved by the Ethics Committee of Dezful University of Medical Sciences, Dezful, Iran (Code: IR.DUMS.REC.1398.040).

### Statistical analysis 

Data analysis was performed in Social Sciences software version 25.0 (SPSS Inc., Chicago, IL, USA). Data were presented as mean ± standard deviation, count, and percentage. Due to a large number of samples and 4 groups in the present study, a one-way ANOVA parametric analysis of variance was used to compare the means in the studied groups, and the Chi-square test was used to compare the relationship between qualitative variables grouped. In order to check the normality of the quantitative data, the non-parametric Kolmogorov–Smirnov test analysis was performed, which showed normal distribution of data (p < 0.05).

## 3. Results

A total of 1246 participants fulfilling the criteria defined in materials and methods participated in this study and received embryo transfer.

The subjects were divided into 4 groups based on the embryo transfer day, including on day 2 (group 1 = 355 (28.5%), on day 3 (group 2 = 571 (45.8%), on day 4 (group 3 = 191 (15.3%) and on day 5 or 6 (group 4 = 129 (10.4%). Table I presents the demographic characteristics of the patients. The mean age of women and men was 33.12 ± 5.6 and 37.57 ± 6.96 yr, respectively. The mean duration of infertility and endometrial thickness 6.59 ± 4.87 yr and 8.02 ± 0.92 mm, respectively. The groups were homogenous regarding patient characteristics. No significant differences were observed among the groups regarding men and women's age, duration of infertility, kind and cause of infertility, frequency of IVF/ICSI cycles, and endometrial thickness (Table I). The most important causes of infertility were a male factor (n = 426), female factor (n = 446), unexplained factor (n = 42), male and female factor (n = 332). Female factors include an ovarian factor (n = 107), and polycystic ovaries (n = 267), tubal factor (n = 32), hypothalamic amenorrhea (n = 40).

Table II shows the participant's cycle characteristics. No significant differences were observed in terms of estradiol levels on the day of HCG injection, the number of follicles, recovered oocytes, the number of metaphase II oocytes, total number of embryos, number of transferred embryos, and score of transferred embryos. According to the findings from a total of 2437 obtained embryos, 1175 embryos (48.2%) had A quality, 703 (28.8%) had B quality, 480 (19.7%) had C quality, 79 (3.3%) had D quality that were eliminated for transfer. The distribution of transferred embryos was the same in all groups.

Table III shows the comparison of fertility and its consequences based on the age of the transferred embryo. No difference was observed between groups regarding chemical and clinical pregnancy rates p = 0.96, p = 0.89, respectively. Regarding ongoing pregnancy and live birth rates, results were indicated despite the lower rates of ongoing pregnancy and live birth in groups 3 and 4, this difference was not significant p = 0.78, p = 0.4, respectively. It was expected that the highest and lowest rates of multiple pregnancies were seen in groups 4 and 1, respectively; however, no significant difference was observed in this regard (p = 0.19). Our findings indicated that no correlation was observed between the developmental stage of transferred embryos and multiple births. According to the obtained results, the highest percentage of abortions was observed in group 3, although this rate was not significantly different from the other groups (p = 0.54).

**Table 1:** Demographic parameters of the study groups


**Variables**		**Group 1 (n = 355)**	**Group 2 (n = 571)**	**Group 3 (n = 191)**	**Group 4 (n = 129)**	**P-value**
Age (yr)*						
	**Female**	30.14 ± 5.21	31.7 ± 3.77	33.43 ± 4.11	32.39 ± 4.83	0.18
	**Male**	35.36 ± 4.3	36.4 ± 4.82	35.97 ± 3.45	37.76 ± 3.65	0.26
Duration of infertility (yr)*		5.24 ± 3.7	6.55 ± 3.98	5.71 ± 4.36	6.08 ± 4.28	0.54
Etiology of infertility**						
	**Male factor**	116 (32.6)	189 (33.1)	78 (40.8)	43 (33.3)	0.86
	**Female factor**	134 (37.8)	210 (36.8)	55 (28.8)	47 (36.4)	0.64
	**Both factor**	94 (26.5)	156 (27.3)	49 (25.7)	33 (25.6)	0.09
	**Unexplained factor**	11 (3.1)	16 (2.8)	9 (4.7)	6 (4.7)	0.08
Kind of infertility**						
	**Primary**	289 (81.4)	461 (80.8)	154 (80.6)	106 (82.2)	0.62
	**Secondary**	66 (18.6)	110 (19.2)	37 (19.4)	23 (17.8)	0.13
Number of IVF/ICSI cycles**						
	**IVF**	109 (30.7)	175 (30.6)	57 (29.9)	38 (29.5)	0.12
	**ICSI**	246 (69.3)	396 (69.4)	134 (70.1)	91 (70.5)	0.57
Endometrial thickness (mm)*		8.02 ± 3.74	7.58 ± 4.13	7.44 ± 4.9	8.15 ± 3.6	0.96
*Data presented as Mean ± SD. One-way ANOVA, **Data presented as n (%), Chi-square test. IVF: In vitro fertilization, ICSI: Intracytoplasmic sperm injection						

**Table 2:** Cycle characteristics of the study groups


**Variables**		**Group 1 (n = 355)**	**Group 2 (n = 571)**	**Group 3 (n = 191)**	**Group 4 (n = 129)**	**P-value**
**Peak estradiol on day of** hCG injection (pg/ml)*		1892.3 ± 6.8	1905 ± 3.91	1957.6 ± 3.67	1986.9 ± 4.78	0.32
Number of follicles ≥ 16 mm*		9.04 ± 4.28	8.36 ± 3.54	7.66 ± 4.31	6.13 ± 3.57	0.68
Number of oocytes*		9.12 ± 2.5	8.62 ± 3.45	8.32 ± 4.65	7.46 ± 2.8	0.74
Number of metaphase II oocytes*		8.25 ± 3.74	6.29 ± 5.19	5.98 ± 3.7	5.79 ± 4.16	0.42
Total embryos*		6.5 ± 3.16	6.08 ± 3.32	4.29 ± 2.49	4.08 ± 2.58	0.46
**Number of transferred embryos** in each group*		2.15 ± 0.74	2.27 ± 0.84	2.33 ± 0.34	1.95 ± 0.67	0.09
Score of transferred embryos**						
	**Grade A**	374 (48.8)	525 (51.4)	164 (50.5)	112 (45.9)	0.67
	**Grade B**	231 (30.1)	302 (29.6)	88 (27)	82 (33.6)	0.43
	**Grade C**	162 (21.1)	195 (19)	73 (22.5)	50 (20.5)	0.2
*Data presented as Mean ± SD. One-way ANOVA test, **Data presented as n (%), Chi-square test, hCG: Human chorionic gonadotropin						

**Table 3:** Pregnancy outcomes and complications in the study group


**Pregnancy outcome**	**Group 1 (n = 355)**	**Group 2 (n = 571)**	**Group 3 (n = 191)**	**Group 4 (n = 129)**	**P-value**
**Chemical pregnancy rate**	108 (30.4)	189 (32)	54 (28.3)	28 (21.7)	0.96
**Clinical pregnancy rate**	75 (21.1)	132 (23.1)	35 (18.3)	22 (17)	0.89
**Ongoing pregnancy rate**	62 (17.4)	115 (20.1)	27 (14.1)	18 (14)	0.78
**Live birth rate**	68 (19.1)	114 (20)	27 (14.1)	18 (14)	0.40
**Multiple pregnancy rate**	10 (2.8)	16 (2.8)	4 (2)	4 (3.1)	0.19
**Miscarriage rate**	13 (3.7)	17 (3)	8 (4.2)	4 (3.1)	0.54
Data presented as n (%). Chi-square test					

## 4. Discussion 

Recent advances in cell culture have led to a shift in IVF from embryo transfer in the initial stages of cleavage to embryo transfer in the blastocyst stage. In terms of blastocyst culture and its transfer theory, this approach could improve the synchrony between the fetus and uterine endometrium. Our findings among 1246 eligible cases indicated no significant correlation between pregnancy outcomes and the embryo's developmental stage in embryo transfer cycles.

Research in participants aged > 36 yr, observed that BET compared to the cleavage stage was more likely to increase the probability of achieving a live birth. Also, they concluded that one good quality blastocyst reduces the likelihood of multiple pregnancies in those aged < 36 yr (11). Contrary to the findings of the mentioned study, the age of embryo transfer did not have a significant correlation with pregnancy outcomes in the current research. This discrepancy could be due to the differences in sample size, population age, and the ratio of the transferred embryos in the cleavage and blastocyst stages.

In a study to investigate the possible impact of COS on the perinatal outcomes in 784 fresh transfers and 382 freeze-thawed dual blastocyst transfers showed clinically significant differences concerning the peri-implantation and perinatal outcomes of fresh and freeze blastocyst transfer, due to better endometrial receptivity and placentation in freeze-thawed cycles (12). This discrepancy could be due to the selection of fresh embryo transfer in this study that can be an effect of COS on implantation rate and pregnancy outcomes.

In another retrospective study of 11,801 cases that underwent cleavage-stage embryo transfer (CSET) and 1009 cases that underwent BET concluded no evidence to support the superiority of BET compared with CSET in a freeze-all treatment scenario about living birth rate or by increasing the risk of preterm delivery in BET (13). The findings of the mentioned study, which have been reviewed in a trial study, are consistent with our findings. Despite the selection of cases with frozen embryo transfer in this study, the results are consistent with our study. In this way, eliminating the effect of COS on implantation as the confounding factor further confirms the results of our study.

In a randomized controlled open label pilot clinical trial research, the investigators showed that the transfer of blastocyst-stage embryos in recipients of donated egg is preferred to a higher clinical pregnancy rate, live birth rate, shorter time to pregnancy, and lower costs to succeed live birth, compared with CSET (14). These findings are in contrast with the results of our study.

One retrospective study about the assessment of the cumulative mean rate of live births in 377 embryos transferred on day 3 and 623 embryos transferred on day 5, showed no significant difference in the rate of live births between the 2 groups (15). This finding is consistent with our results that showed the rate of live birth in the cases where 2 days old embryo was estimated at 19.1%, while it was 20% with 3 days old embryo, 14.1% with 4 days, and 14% with 5 or 6 days; however, the difference in this regard was not considered significant.

The results of the one retrospective cohort study show that embryo transfer in the blastocyst stage increases the chance of live-birth rate by 1.49 times. At the same time, it also increases the probability of preterm birth by 16%. The probability of clinical pregnancy also increases by 1.68 times (16). These results are not consistent with our results in this research; the average rate of live births at the cleavage and blastocyst stages was 17.6% and 14%, respectively. However, the increase in the fertility rate did not change significantly. The fertility rate based on the fetal heart development was estimated at 20.6% for the embryos in the cleavage stage and 17% in the blastocyst stage.

Also, in contrast to these studies, the results of our study showed low ongoing pregnancy and live-birth rate on the 4 ${}^{\text{th}}$ and 5 ${}^{\text{th}}$ -6 ${}^{\text{th}}$ days embryo groups than on the 2 ${}^{\text{nd}}$ and 3 ${}^{\text{rd}}$ day's embryo groups, although the difference was not significant. The allocation of more samples with BET in these researches than in the present study is one of the reasons for the difference in the results obtained in the 2 studies.

On the other hand, research about live birth following blastocyst against CSET in the first cycle of IVF, results showed blastocyst transfer did not significantly affect the odds of live birth (17) that no consistent with our results in this regard. Also, findings of other studies showed that there is no significant difference between blastocyst and CSET. However, embryo transfer in the blastocyst stage is associated with a reduced likelihood of multiple pregnancies (2). In this regard, although the rate of twin pregnancies did not decrease in our study, it did not differ significantly compared to other groups. The difference in the number and quality of embryos transferred as well as the choice of patients can probably be justified.

### Limitations and advantages

One of the limits of our analysis includes the retrospective kind. We were incapable of adjusting for several confounders, such as smoking status, body mass index. Also, this study failed to assess the cumulative live birth rate as the most valuable key performance indicator of the treatment. Also, perinatal outcomes were not included in our analyses. Advantage of the study we were able to assess the various stages of cleavage fresh embryo transfer and blastocyst stage on pregnancy outcomes. Also, all processes from the choice of cases, indications, IVF procedure, ovum pick up, and embryo transfer were done by one gynecologist that confounder caused by doing, was deleted.

## 5. Conclusion

According to the results, the developmental stage of the fresh embryo (blastocyst or cleavage stage) for transfer had no positive or negative effect on fertility. It seems BET is not superior to cleavage embryo transfer in reproductive outcomes. However, it is suggested that similar investigations can be conducted in this regard on larger sample sizes to assess the different stages of embryo transfer, pregnancy, and perinatal outcomes.

##  Conflict of Interest 

Authors declare that there is no conflict of interest.
